# Coverage of diabetes complications screening in rural Eastern Cape, South Africa: A cross-sectional survey

**DOI:** 10.4102/safp.v64i1.5447

**Published:** 2022-04-25

**Authors:** Eyitayo O. Owolabi, Daniel T. Goon, Anthony I. Ajayi, Oladele V. Adeniyi, Kathryn M. Chu

**Affiliations:** 1 Centre for Global Surgery, Department of Global Health, Faculty of Medicine and Health Sciences, Stellenbosch University, Cape Town, South Africa; 2 Department of Public Health, Faculty of Health Sciences, University of Fort Hare, East London, South Africa; 3 Population Dynamics and Sexual and Reproductive Health Unit, African Population and Health Research Centre, Nairobi, Kenya; 4 Department of Family Medicine, Faculty of Health Sciences, Walter Sisulu University/Cecilia Makiwane Hospital, East London, South Africa

**Keywords:** diabetes, primary healthcare, screening, diabetes-related complications, South Africa

## Abstract

**Background:**

There is a paucity of data on the coverage of diabetes mellitus (DM) complications screening in primary healthcare facilities in South Africa (SA). This study assesses the extent of screening for DM complications among individuals with type 2 DM attending primary health facilities in rural Eastern Cape (EC), SA.

**Methods:**

The study adopted a descriptive, cross-sectional design and obtained data from 372 individuals with type 2 diabetes attending six selected primary healthcare centres (PHCs) in two EC districts. Demographic and clinical data were obtained through questionnaire-based interviews and reviews of medical records. We assessed the extent of screening for estimated glomerular filtration rate (eGFR), fasting lipogram, eye examination, foot examination and glycated hemoglobin (HbA1c) in the past year.

**Results:**

Participants mean age was 62 (standard deviation [s.d.] ± 11) years, and their mean duration of diagnosis was 9 (s.d. ± 8) years. In the past year, HbA1c result was available for 71 (19.1%) of the participants; 60 (16.1%) had eGFR results, while only 33 (8.9%) had documented lipid results. In total, 52 (14.0%) had carried out eye examinations, while only 9 (2.3%) had undergone foot examinations in the past year. About two-thirds of the participants (59.9%) had not undergone any form of complication screening in the past year, and none had undergone the complete screening panel.

**Conclusion:**

The coverage of screening for DM complications was low across all indicators. Studies to understand barriers to and facilitators of DM complications screening at PHCs are required. Also, interventions to improve diabetes complication screening in the region are needed and should target the primary healthcare providers.

## Background

Diabetes mellitus (DM) is a serious public health concern associated with significant morbidity, mortality and disability.^1^ In South Africa (SA), 12.8% of the adult population lives with diabetes,^2^ and a significant proportion of them have uncontrolled diabetes, compounded by the presence of other comorbidities.^3,4,5^ Consequently, individuals are predisposed to microvascular and macrovascular complications, resulting in a reduced quality of life, an increased risk of premature mortality and increased healthcare expenditure.^6,7^

Treatment guidelines, including those of the Society of Endocrinology, Metabolism and Diabetes of South Africa (SEMDSA) and the South African Primary Health Care guidelines, strongly emphasise the need for regular screening for diabetes complications to improve treatment outcomes.^8,9^ Specifically, in this study setting, individuals with type 2 DM are provided with a monthly drug supply and are expected to visit the clinic periodically for drug refills and assessments in the absence of urgent medical conditions. At each of these visits, it is recommended that weight, body mass index for cardiovascular risk if appropriate, blood glucose and blood pressure measurements be carried out during patients’ assessments by nurses or nursing assistants. Also, screening for diabetic kidney disease should be performed at diagnosis and subsequently on an annual basis for individuals with type 2 DM using the urine albumin-to-creatinine ratio and estimated glomerular filtration rate (eGFR). Individuals with type 2 DM should be screened for diabetic retinopathy at diagnosis and then annually, or in resource-limited areas every two years, provided the blood glucose level is controlled. Likewise, foot examinations are recommended annually or more frequently among those at risk of developing foot ulcers.^8,9^

The South African public healthcare system serves 84% of the population,^10^ with 69% of those in the lowest socio-economic quantile mainly using the primary healthcare centres (PHCs).^11^ Many people living with diabetes and other chronic illnesses are managed at the PHCs.^12^ The primary healthcare providers play a central role in implementing promotive and preventive aspects of healthcare.^12^ The management of end-organ complications from DM, such as chronic renal failure, retinopathy and foot infections, may require specialised care, which is usually found at the hospitals. If PHCs fail to conduct thorough screenings and make prompt referrals when needed,^8,13,14^ individuals with complications will be missed, and treatment will be delayed, resulting in increased healthcare costs, poor prognosis and mortality.^15^ Therefore, it is imperative to assess the extent of diabetes complications screening at PHCs, where most people receive health care. Regular screening of patients with diabetes to promptly identify complications is also a marker of the quality of care received at the PHCs.

The extent of screening for DM complications has been assessed in three of the 11 provinces in SA^4,5,16^ but only one of the studies was conducted at the PHC level.^16^ Webb et al.^16^ reported that the fasting glucose test was conducted for only 1.5% of the patients, reported HbA1c for 23% of patients, lipogram or total cholesterol for 26%, while 21% of the patients were assessed for kidney function using serum creatinine levels and 60% of them were assessed using the urine dipstick. They further reported that only 8% had had an eye assessment and 6% had had their foot assessed in the preceding year. All these studies are now over 6 years and may not reflect the current situation. Also, there are no available data on the level of screening for diabetes complications in the Eastern Cape (EC), one of the poorest provinces in SA, with a high prevalence of diabetes and poor glycaemic control level.^3,17^ Therefore, this study aimed to assess the extent of screening for diabetes-related complications among individuals with type 2 diabetes at selected PHCs in the rural EC province, SA. This information is vital for informing health policy decisions and advocacy. In addition, findings could provide necessary data for strengthening the health system.

## Methods

### Study design and settings

This descriptive, cross-sectional study was conducted among individuals with type 2 diabetes in the EC province, SA. The EC province was created in 1994, comprising the old Xhosa ‘homelands’ of the Transkei and Ciskei and part of the Cape province. The EC province is one of the poorest provinces in SA.^18,19^ The province comprises two metropolitan municipalities: Buffalo City and the Nelson Mandela Bay Metropolitan Municipalities, and six districts: Alfred Nzo, Amathole, Chris Hani Joe Gqabi, OR Tambo and Sarah Baartman.^19^

This was a sub-study of a larger study, which sought to determine the effectiveness of an mHealth intervention that aimed at improving glycaemic control, conducted between July 2018 and April 2019.^17^ This study was conducted at two purposively selected districts of the eight health districts in EC province: Buffalo City Metropolitan Municipality and Amathole District. Guidelines for the management of diabetes are the same across all the primary healthcare facilities in SA.^8^ In each of these health districts, three PHCs were conveniently selected, bringing the total to six PHCs.

Primary health care in SA is provided through a nurse-based, doctor-supported infrastructure of clinics and community health centres (CHCs), available within 5 km of home for more than 90% of the population and free at the point of use. Clinics are smaller health facilities, staffed by nurses and with sessional visiting doctors (usually 4 h – 8 h a week). Community health centres are larger facilities that are staffed by a multidisciplinary PHC team consisting of nurses, doctors, pharmacists and allied health workers. Individuals with diabetes are usually seen by nurses who assess their blood glucose level and blood pressure and prescribe medications following the treatment guidelines. Those with a poorly controlled glycaemic status are seen by doctors for further management. For urgent and more complicated cases, patients may be referred directly to the nearest hospitals.

### Sample and sampling technique

The sample size for this study was estimated based on the 23% reported rate of screening for HbA1c by Webb et al.^16^ using the formula:

$$\text{n}={\text{z}}^{2}*\text{p}*(1-\text{p})/{\text{e}}^{2},$$ [Eqn 1]

where z = 1.96 for a confidence level (α) of 95%, p = proportion (expressed as a decimal), e = margin of error.

$$\begin{array}{l} \text{Z}=1.96,\text{p}=0.23,\text{e}=0.45\\ \text{n}=1.96^{2}\times 0.23*(1-0.23)/0.045^{2}\\ n=336. \end{array}$$ [Eqn 2]

The estimated sample size was 336, adjusted by 15% to account for incomplete data.

All ambulatory DM individuals who met the eligibility criteria and were willing to participate were recruited consecutively at the selected clinics. Primary health care centres with specific diabetes clinics were visited on scheduled days, while those which run open clinics were visited daily. Data collection was carried out for a minimum of two weeks at each clinic.

### Eligibility criteria

Participants were included if previously diagnosed with type 2 diabetes, aged ≥ 18 years, attending the outpatient clinics of the selected PHCs, and if willing to participate. All those who were critically ill were excluded from the study and directed for acute care management.

### Data collection

The primary investigator (EOO), who is a professional nurse, conducted interviews and reviewed medical records of patients. Socio-demographic variables included gender, age, education level, marital status, average monthly income and employment status. Clinical characteristics assessed were as follows: type of diabetes, year of diagnosis, type of treatment and comorbidity.

Data on screening for DM complications in the past 12 months were obtained through a review of medical records. These included eGFR and dipstick urinalysis for albumin to ascertain kidney function, fasting lipids, eye examination for cataract and retinopathy, foot examination for diabetic foot (infections, peripheral vascular disease and neuropathy), and HbA1c for glycaemic status. Patient self-reporting was used where information on non-laboratory-based complication screening was absent in the medical records. Specifically, patients were asked about eye and foot examinations in the previous 12 months. Responses to these questions were either ‘Yes’ or ‘No’. In addition, random glucose checks were carried out during the interviews.

The data collection tool was piloted with 20 participants at one of the clinics to determine its validity and feasibility and was revised as necessary. The results from the pilot study were excluded from the data analysis.

### Ethical considerations

The Research Ethics Committee of the University of Fort Hare granted ethical approval (reference number: GOO171OWA01) for this study. We also obtained approvals from the EC Department of Health, the two selected health districts and the clinic heads. After detailed information, written informed consents were obtained from the participants before the commencement of the study. Rights to anonymity and confidentiality were ensured during and after the study, and participants consented to referral for further care in case of detection of abnormal findings.

### Data analysis

After data capturing, data were checked for completeness and accuracy. Prior to analysis, data on individuals with type 1 diabetes (*n* = 27) were excluded, and final analysis was conducted on individuals with type 2 diabetes. Descriptive statistics were used to describe the proportion of participants who had undergone any or all of the complications screening. Data were expressed as counts (frequency) and proportions (%) for categorical variables, while mean values (±Standard deviation [s.d.]) were used to summarise continuous variables. Percentages were compared using the Chi-square (χ^2^) test. A *p*-value of < 0.05 was considered statistically significant. All statistical analyses were performed using IBM Statistical Package for Social Science (SPSS) version 25 for Windows (IBM Corps, Armonk, New York, United States [US]).

## Results

There were 372 participants, including 306 (82.3%) females. All were black Africans and 306 (82.3%) were unemployed. The mean age was 62 (s.d. ± 11) years, and the mean duration of DM diagnosis was 9 (s.d. ± 8) years. The mean monthly income was R1857 (s.d. ± 1868) and ranged from R150.00 to R18 000.00. About three-quarters of the participants used only oral DM medication, that is, 295 (79.3%), while 47 (12.6%) used only insulin (Table 1).

**TABLE 1:** Participants’ characteristics.

Variables	Frequency (*n*)	Percentage (%)
**Gender**		
Male	66	17.7
Female	306	82.3
**Highest level of education**		
No formal education	9	2.4
Grade 1–7	148	39.9
Grade 8–12	195	52.6
Tertiary	8	2.2
Post-grad	11	3.0
**Marital status**		
Never married	88	23.9
Married	170	46.2
Single mother	7	1.9
Divorced	16	4.3
Widowed	86	23.4
Cohabiting	1	0.3
**Employment status**		
Government employee	7	1.9
Non-government employee	13	3.5
Self-employed	10	2.7
Retired	36	9.7
Unemployed	306	82.3
**Average monthly income (rand)**		
R0.00 – R1500.00	51	16.6
R1501.00 – R3000.00	236	76.9
More than R3000.00	20	6.5
**Duration of diabetes diagnosis (years)**		
10 or less	262	70.6
More than 10	109	29.4
**Duration of diabetes treatment (years)**		
10 or less	262	70.6
More than 10	109	29.4
**Type of treatment**		
Oral pills	295	79.3
Insulin	47	12.6
Both	30	8.1
**Fasting blood sugar (mmol/L)**		
< 10	131	35.2
≥ 10	241	64.8
**Blood pressure (mmHg)**		
< 140/90	126	34.1
≥ 140/90	244	65.9

The rates of screening for complications among the patients in the past year are presented in Table 2. HbA1c screening had been conducted for only 71 (19.1%) of the participants. Also, 60 (16.1%) had undergone tests for kidney function. Only eight (2.2%) patients had undergone a foot examination in the past year.

**TABLE 2:** Screening for complications among participants in the past 12 months.

Variable	Yes		No	
	*N*	%	*n*	%
HBA1c	71	19.1	301	80.9
eGFR	60	16.1	312	83.9
Lipids	33	8.9	339	91.1
Eyes screening	52	14.0	320	86.0
Foot examination	8	2.2	364	97.8
Urine test	30	8.1	342	91.9

HBA1c, glycated hemoglobin; eGFR, estimated glomerular filtration rate.

**FIGURE 1:**
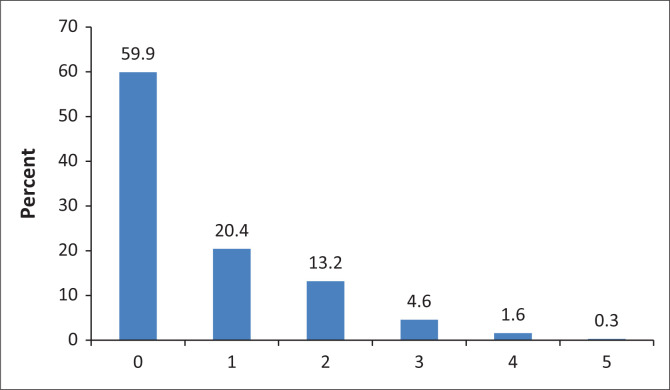
Number of screenings carried out.

Also, 223 (59.9%) of the participants had not undergone any form of complication screening in the past year, and none had the complete screening panel (Figure 1).

As shown in Table 3, HbA1c testing was significantly associated with random blood glucose (RBG) levels and health facility types. HbA1c testing was higher among participants with RBG ≥ 10 mmol/L (22.8%) than among those whose RBG was < 10 mmol/L (12.2%, *p* = 0.008). Also, the rate of HbA1c testing among participants managed at CHCs was higher (22.8%) than among those who were managed at the clinics (12.6%, *p* = 0.010). We also found a significant association between eGFR testing and RBG levels (*p* = 0.046). The eGFR testing rate was higher among patients with RBG ≥ 10 mmol/L (18.7%) than among those with RBG < 10 mmol/L (11.5%). Likewise, eye screening was associated with treatment type (*p* = 0.009). The rate of eye screening was higher among those managed with both insulin and oral pills (26.7%) than among those managed with insulin only (23.4%) or oral pills only (11.2%).

**TABLE 3:** Screening for complications stratified by demographic and clinical variables.

Variable	HbA1c test		eGFR		Lipids		Eye screening		Foot examination		Urine testing	
	*n*	%	*n*	%	*n*	%	*n*	%	*n*	%	*n*	%
**Level of education**												
Grade 7 and below	36	22.9	31	19.7	11	7.0	22	14.0	5	3.2	17	10.8
Grade 8 and above	35	16.4	29	13.6	22	10.3	30	14.0	3	1.4	13	6.1
**Employment status**												
Employed	7	23.3	6	20.0	5	16.7	7	23.3	0	0.0	1	3.3
Unemployed	64	18.7	54	15.8	28	8.2	45	13.2	8	2.3	29	8.5
**Average monthly income**												
0–1500	6	11.8	4	7.8	3	5.9	8	15.7	0	0.0	4	7.8
More than 1500	50	19.5	46	18.0	21	8.2	35	13.7	6	2.3	21	8.2
**Typ e of treatment**												
Oral pills	53	18.0	47	15.9	26	8.8	33*	11.2	5	1.7	22	7.5
Insulin	12	25.5	8	17.0	5	10.6	11	23.4	2	4.3	6	12.8
Both	6	20.0	5	16.7	2	6.7	8	26.7	1	3.3	2	6.7
**Hypertension history**												
Yes	61	19. 2	54	17.0	30	9.4	48	15.1	8	2.5	29	9.1
No	10	18.5	6	11.1	3	5.6	4	7.4	0	0.0	1	1.9
**Random blood glucose (mmol/L)**												
< 10	16	12.2*	15	11.5*	9	6.9	22	16.8	4	3.1	8	6.1
≥ 10	55	22.8	45	18.7	24	10.0	30	12.4	4	1.7	22	9.1
**Health facility type**												
Primary health clinics	17	12.6*	17	12.6	14	10.4	20	14.8	4	3.0	7	5.2
Community health clinics	54	22.8	43	18.1	19	8.0	32	13.5	4	1.7	23	9.7
**Diabetes duration**												
≤ 10 years	52	19.8	39	14.9	22	8.4	36	13.7	6	2.3	21	8.0
≥ 10 years	19	17.4	21	19.3	11	10.1	16	14.7	2	1.8	9	8.3

HBA1c, glycated hemoglobin; eGFR, estimated glomerular filtration rate.

* , *p*-value < 0.05.

## Discussion

While South African national guidelines recommend annual kidney, eye and foot screenings among individuals with type 2 DM for microvascular and macrovascular complications, this study has demonstrated that the selected PHCs in the two districts in a rural province fell short of this target. The efficacy and cost-effectiveness of annual screenings in DM complications prevention are well-documented.^20,21^ The health and socio-economic impacts of DM complications are enormous^6,7,22^, and the low extent of screening for these complications in our setting is of great concern. With such a low level of complications screening, many individuals with DM may develop preventable complications, which may be detected too late for possible interventions.^23^ Although we did not explore the reasons for such low coverage of complications screening in this setting, similar findings have been reported in another SA province.^16^ Perhaps, the physical and human resources required for such screenings are very limited in the study settings. New models of healthcare such as mhealth may be considered to offer specialised screening services for persons with type 2 diabetes. Also, some evidence-based guideline adjustments may be required. For instance, eyes examination every 1–2 years following one or more eye examinations and for those with well-controlled diabetes may be more feasible or cost-effective. Agardh et al.^24^ showed that 3-year retinal screening intervals can be recommended for persons with type 2 diabetes and with no retinopathy.

Glycated hemoglobin is the gold standard measure for glycaemic control, and routine biannual testing is recommended for those with good glycaemic control.^8,9^ Also, HbA1c testing is required to assess patients’ management, design new treatment plans and evaluate progress. For this reason, HbA1c testing is also recommended every three months after every treatment change or in uncontrolled DM. Yet, only 19.5% of the patients had undergone HbA1c testing in this setting, indicating that patients’ glycaemic status had been poorly monitored. As such, blood glucose may deteriorate, without healthcare workers’ knowledge, in patients whose levels were previously under control, as well as in patients whose blood glucose is currently uncontrolled. Without such testing, those on new treatment regimens may also deteriorate because of the lack of adequate monitoring.

It is critical to emphasise the low coverage of screening for complications in individuals with type 2 DM as reflected by surrogate markers of retinopathy, nephropathy, neuropathy and others in this study. These findings reflect the quality of services currently offered at the PHCs in the region, and therefore, call for urgent action. The SEMDSA and the South African Primary Health Care guidelines^8,9^ have provided guidance on the frequency of screening for DM complications. Health authorities should now focus on the effective implementation of these evidence-based guidelines in order to improve the quality of care and health outcomes in individuals with DM in the region.

While this study did not measure barriers to routine DM complication screening, several possible reasons such as physical and human resource constraints may explain the study findings.^5^ For instance, we found significantly higher rates of HbA1c testing among those attending care at CHCs than among those receiving care at the clinics. This may be because CHCs have more healthcare providers, including doctors, nurses and others, along with more infrastructural resources. Training and delegation of community health workers or lesser qualified care providers to conduct some screenings may be one way forward. This has been shown to be effective for DM retinopathy screening.^25^ While this is a plausible reason, it is important to ascertain specific reasons for such a low level of screening in this setting to design appropriate interventions.

### Strengths and limitations

The cross-sectional nature of this study and convenience sampling are apparent limitations. The use of self-reporting might also be associated with recall bias. However, we mitigated this limitation through the review of records to confirm the information given. Also, only a few clinics were covered; thus, results may not be generalisable to the entire province. Moreover, in some instances, patient records lacked complete information which might have impacted the accuracy of the results. Despite these limitations, the methodological rigour of verifying self-reported information with a thorough perusal of all available records and laboratory results are important strengths of this study. Also, the information gathered will serve as a reference point for issues regarding compliance to treatment guidelines for diabetes in the EC province.

## Conclusion

Screening for DM complications in rural South African primary health clinics is very low. There is a need for implementing measures to improve patient screening and adherence to evidence-based guidelines for improved diabetes care, management and outcomes in the setting. South Africa operates a tiered public healthcare system. The primary healthcare facilities are most accessible to the majority of the population. Access to quality health care is essential for promoting health care and improving health outcomes. Therefore, it is critical for services such as prompt screening for complications and proper disease management to be available at this level of care. Future studies should ascertain possible reasons for such a low level of screening for complications in order to guide the development of appropriate interventions.

## References

[1] World Health Organization. Global report on diabetes [homepage on the Internet]. 2016 [cited 2020 May 08]. Available from: https://apps.who.int/iris/bitstream/handle/10665/204871/9789241565257_eng.pdf;jsessionid=EB3E971A8CB481720E6CA32C262B5026?sequence=1

[2] Federation ID. Diabetes in South Africa [homepage on the Internet]. 2019 [cited 2020 May 08]. Available from: https://idf.org/our-network/regions-members/africa/members/25-south-africa.html

[3] Adeniyi OV, Yogeswaran P, Longo-Mbenza B, Ter Goon D, Ajayi AI. Cross-sectional study of patients with type 2 diabetes in OR Tambo district, South Africa. BMJ Open. 2016;6(7):e010875. 10.1136/bmjopen-2015-010875 PMC4986079 27473948

[4] Igbojiaku OJ, Ross A, Harbor OC. Compliance with diabetes guidelines at a regional hospital in KwaZulu-Natal, South Africa. Afr J Prim Health Care Fam Med. 2013;5(1):1–5. 10.4102/phcfm.v5i1.447

[5] Tumbo JM, Kadima FN. Screening of long-term complications and glycaemic control of patients with diabetes attending Rustenburg Provincial Hospital in North West Province, South Africa. Afr J Prim Health Care Fam Med. 2013;5(1):a375. 10.4102/phcfm.v5i1.375

[6] Asif M. The prevention and control the type-2 diabetes by changing lifestyle and dietary pattern. J Educ Health Promot. 2014;3:1. 10.4103/2277-9531.127541 24741641 PMC3977406

[7] Nolan CJ, Damm P, Prentki M. Type 2 diabetes across generations: From pathophysiology to prevention and management. Lancet. 2011;378(9786):169–181. 10.1016/S0140-6736(11)60614-4 21705072

[8] Amod A, Berg G. The 2012 SEMDSA guidelines for the management of type 2 diabetes. J Endocrinol Metab Diabetes S Afr. 2012;17(2):S4. 10.1080/22201009.2012.10872276

[9] South African Department of Health. Primary care guideline [homepage on the Internet]. 2015 [cited 2021 June 11]. Available from: https://health-e.org.za/2015/05/07/guidelines-primary-care-101/

[10] National Department of Health. National Health Insurance for South Africa: Towards universal health coverage. [homepage on the Internet]. 2015 [cited 2021 June 28]. Available from: https://www.gov.za/sites/default/files/gcis_document/201512/39506gon1230.pdf

[11] Harris B, Goudge J, Ataguba JE, et al. Inequities in access to health care in South Africa. J Public Health Policy. 2011;32(1):S102–S123. 10.1057/jphp.2011.35 21730985

[12] Pouane T. South African health review [homepage on the Internet]. 2008 [cited 2021 May 08]. Available from: https://www.hst.org.za/publications/South%20African%20Health%20Reviews/sahr2008.pdf

[13] Dookie S, Singh S. Primary health services at district level in South Africa: A critique of the primary health care approach. BMC Fam Pract. 2012;13(1):1–4. 10.1186/1471-2296-13-67 22748078 PMC3403923

[14] Hewapathirana N, Page S. Diabetic microvascular complications – Screening, diagnosis and prevention. Clin Focus Prim Care. 2012;6(3):177–191.

[15] Li R, Bilik D, Brown MB, et al. Medical costs associated with type 2 diabetes complications and comorbidities. Am J Manag Care. 2013;19(5):421. 23781894 PMC4337403

[16] Webb EM, Rheeder P, Van Zyl DG. Diabetes care and complications in primary care in the Tshwane district of South Africa. Prim Care diabetes. 2015;9(2):147–154. 10.1016/j.pcd.2014.05.002 24933340

[17] Owolabi EO, Goon DT, Ajayi AI. Efficacy, acceptability and feasibility of daily text-messaging in promoting glycaemic control and other clinical outcomes in a low-resource setting of South Africa: A randomised controlled trial. PLoS One. 2019;14(11):e0224791. 10.1371/journal.pone.0224791 31774842 PMC6881007

[18] Business Tech. The richest and the poorest municipalities in South Africa [homepage on the Internet]. 2016 [cited 2021 June 28]. Available from: https://businesstech.co.za/news/wealth/127213/the-richest-and-poorestmunicipalities-in-south-africa/

[19] Stats SA. South African statistics, 2011 [document on the Internet]. 2011 [cited 2021 May 08]. Available from: http://www.statssa.gov.za/publications/SAStatistics/SAStatistics2011.pdf

[20] Alavi A, Sibbald RG, Mayer D, et al. Diabetic foot ulcers: Part I. Pathophysiology and prevention. J Am Acad Dermatol. 2014;70(1):1.E1–1.E18. 10.1016/j.jaad.2013.06.055 24355275

[21] Scanlon PH. The English national screening programme for diabetic retinopathy 2003–2016. Acta Diabetol. 2017;54(6):515–525. 10.1007/s00592-017-0974-1 28224275 PMC5429356

[22] Armstrong DG, Wrobel J, Robbins JM. Guest editorial: Are diabetes-related wounds and amputations worse than cancer. Int Wound J. 2007;4(4):286–287. 10.1111/j.1742-481X.2007.00392.x 18154621

[23] Adeniyi OV, Owolabi EO. Cross-sectional study of diabetes kidney disease in the Eastern Cape, South Africa. Medicine. 2020;99(50):e23303. 10.1097/MD.0000000000023303 33327258 PMC7738037

[24] Agardh E, Tababat-Khani P. Adopting 3-year screening intervals for sight-threatening retinal vascular lesions in type 2 diabetic subjects without retinopathy. Diabetes Care. 2011;34(6):1318–1319. 10.2337/dc10-2308 21562322 PMC3114331

[25] Allen ML, Van der Does AMB, Gunst C. Improving diabetic foot screening at a primary care clinic: A quality improvement project. Afr J Prim Health Care Fam Med. 2016;8(1):1–9. 10.4102/phcfm.v8i1.955 PMC5062026 27608673

